# Combination of a Synthetic Bioceramic Associated with a Polydioxanone-Based Membrane as an Alternative to Autogenous Bone Grafting

**DOI:** 10.3390/biomimetics9050284

**Published:** 2024-05-10

**Authors:** Paula Buzo Frigério, Juliana de Moura, Letícia Pitol-Palin, Naara Gabriela Monteiro, Carlos Fernando Mourão, Jamil Awad Shibli, Roberta Okamoto

**Affiliations:** 1 Department of Diagnosis and Surgery, São Paulo State University (UNESP), School of Dentistry, Araçatuba 16015-050, Brazil; paula.frigerio@outlook.com (P.B.F.); juliana.moura032@outlook.com (J.d.M.); leticia.p.palin@unesp.br (L.P.-P.); naara.monteiro@unesp.br (N.G.M.); 2 Department of Periodontology, Tufts University School of Dental Medicine, Boston, MA 02111, USA; 3 Department of Periodontology, Dental Research Division, Guarulhos University, Guarulhos 07023-070, Brazil; jshibli@ung.br; 4 Department of Basic Sciences, São Paulo State University (UNESP), School of Dentistry, Araçatuba 16066-840, Brazil; roberta.okamoto@unesp.br

**Keywords:** bone substitutes, bioceramics, bone grafting, guided bone regeneration, guided tissue regeneration, synthetic polymer

## Abstract

The purpose of this study was to evaluate the repair process in rat calvaria filled with synthetic biphasic bioceramics (Plenum^®^ Osshp-70:30, HA:βTCP) or autogenous bone, covered with a polydioxanone membrane (PDO). A total of 48 rats were divided into two groups (*n* = 24): particulate autogenous bone + Plenum^®^ Guide (AUTOPT+PG) or Plenum^®^ Osshp + Plenum^®^ Guide (PO+PG). A defect was created in the calvaria, filled with the grafts, and covered with a PDO membrane, and euthanasia took place at 7, 30, and 60 days. Micro-CT showed no statistical difference between the groups, but there was an increase in bone volume (56.26%), the number of trabeculae (2.76 mm), and intersection surface (26.76 mm^2^) and a decrease in total porosity (43.79%) in the PO+PG group, as well as higher values for the daily mineral apposition rate (7.16 µm/day). Histometric analysis presented material replacement and increased bone formation at 30 days compared to 7 days in both groups. Immunostaining showed a similar pattern between the groups, with an increase in proteins related to bone remodeling and formation. In conclusion, Plenum^®^ Osshp + Plenum^®^ Guide showed similar and sometimes superior results when compared to autogenous bone, making it a competent option as a bone substitute.

## 1. Introduction

Osseointegrated implants have revolutionized dentistry, improving the function and aesthetics of edentulous patients. However, solutions to prevent peri-implant loss are still required since systemic and physiological factors, fractures, and trauma can lead to bone deficiency and osseointegration failure [1,2]. Guided bone regeneration (GBR) acts as a support framework, thus allowing the titanium implant to osseointegrate into the bone tissue, promoting stability and support for occlusal loads and leading to an excellent clinical prognosis [1].

The osteoconductive, osteoinductive, and osteogenic properties of autogenous grafts make them the gold standard for the repair of bone defects and dehiscences. However, their use has limitations, such as the extensive operative time, unavailability of a donor area, and risk of sequelae for the patient and others [3,4,5,6,7,8,9,10,11]. With this in mind, researchers have sought bone substitutes for autogenous grafts that are capable of reconstructing areas of bone destruction, with synthetic grafts being the evolution in this regard [5,6,11,12]. Synthetic biomaterials have osteoinductive and osteoconductive capacities, so they stimulate growth factors and induce the differentiation of bone cells that are capable of forming new bone [13,14].

Among the best options are synthetic bioceramic materials, which are biocompatible, non-toxic, resorbable, and positively stimulate bone regeneration, forming bone that covers areas of loss [13,15]. Beta-tricalcium phosphate (β-TCP) allows a slight regeneration of bone tissue through cell stimulation, has an ordered action between osteoblastic and osteoclastic cells, and its porous structure, similar to trabecular bone, facilitates vascularization and osteogenesis, causing cell migration and proliferation to occur [5,8,15], but it has low mechanical strength [3,14,15,16]. Hydroxyapatite (HA) has broad osteoconductivity and is less resorbed due to the high composition of calcium and phosphate in its structure (Ca/P), so this biomaterial is resorbed slowly, which gives it high load-bearing strength [8,12,14,16].

β-TCP has rapid degradation, providing a regenerative characteristic, since when it is resorbed, it is quickly replaced by new bone tissue, while HA is less soluble, thus maintaining the built-up region for a longer period [8,11,17,18]. In this way, the bone regeneration properties of both biomaterials are improved when they are combined, as one can make up for the deficiency of the other [4,5,11,12,14].

Synthetic membranes are an attractive resource for tissue regeneration (TGR) and bone regeneration (GBR), as they can support the volume of implanted bone tissue and have a favorable aesthetic result [1,19,20]. To allow osteoblastic migration and stimulate tissue regeneration, these membranes must be biocompatible and resorbable, allow tissue vascularization, and have a mechanical barrier function, promoting space maintenance [19,20,21]. In this way, fully synthetic membranes composed of the polymer poly(dioxanone) (PDO) are often used in surgeries to repair areas of intraosseous defects, periodontal and peri-implant soft tissues, as they allow bone cells to migrate and facilitate the replacement of grafted areas with the formation of healthy bone, as well as acting as a mechanical barrier [16,21,22,23]. This justifies its use in this study as a covering material and to keep the biomaterial in position.

The main objective of the present study was to evaluate the bone repair process in a defect created in a rat calvaria using a new synthetic bioceramic based on hydroxyapatite (HA, 70%) and β-tricalcium phosphate (β-TCP, 30%) covered by a polydioxanone membrane (Plenum^®^ Guide), as a future bone substitute for autogenous grafts.

## 2. Materials and Methods

### 2.1. Animals

The Ethics Committee on the Use of Animals (CEUA) of the Faculty of Dentistry of Araçatuba approved the study, under registration number 028/2021. All experiments were carried out according to the requirements established by ISO 10993: “Biological evaluation for medical devices—Part 6: Tests for local effects after implantation” [24].

Forty-eight adult male rats (*Rattus norvegicus albinus*, Wistar), with an average weight of 300 g and an average age of three months, were used to create the critical defects in the calvaria. The rats were supplied by the animal house of the Universidade Estadual Paulista “Júlio de Mesquita Filho”–FOA/UNESP of Araçatuba. The rats were reserved in boxes, and food and water were provided ad libitum, at an ambient temperature of 22 ± 2 °C, with a 12-h light–dark cycle.

The forty-eight rats were divided into two groups, *n* = 24 animals per group: (1) AUTOPT+PG—particulate autogenous bone covered by a polydioxanone membrane (PDO) (Plenum^®^ Guide) (M3 Health Indústria Comércio de Produtos Médicos Odontológicos e Correlatos S.A., Jundiaí, SP, Brazil) and (2) PO+PG—Plenum^®^ Osshp ([synthetic bone graft (70:30, hydroxyapatite:β-tricalcium phosphate) M3 Health Indústria Comércio de Produtos Médicos Odontológicos e Correlatos S.A., Jundiaí, SP, Brazil]) covered by a polydioxanone membrane (PDO) (Plenum^®^ Guide) (M3 Health Indústria Comércio de Produtos Médicos Odontológicos e Correlatos S.A., Jundiaí, SP, Brazil).

### 2.2. Sample Calculation

The sample distribution was based on data from a study published in 2022 by Pitol-Palin et al. [16] on critical defects in rat calvaria, using autogenous bone or synthetic biomaterial as a bone substitute. Therefore, for this study, the power of the test was calculated using the website: http://www.openepi.com/SampleSize/SSMean.htm (OpenEpi, Version 3, open-source calculator, accessed on 10 November 2023), based on data regarding volume percentage bone (BV.TV), in which the mean and standard deviation of group Y = 82.7 ± 6.7 and group Z = 84.3 ± 6.3 with a significance level of 5% and power of 95% in a one-tailed hypothesis test; this suggested a sample size lower than what the present study proposed.

### 2.3. Surgical Procedure (Critical Calvaria Defect)

**Figure 1:**
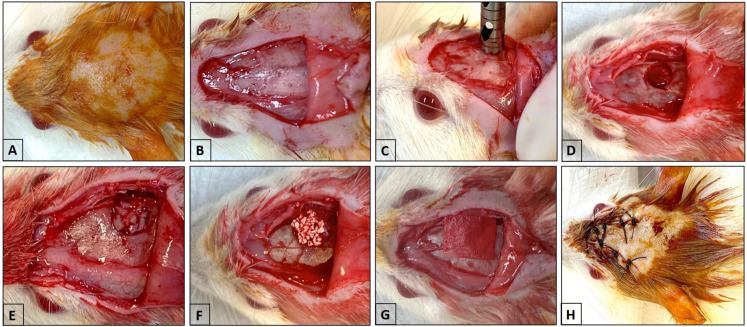
Surgery to create a critical defect in the calvarium. (**A**) Trichotomy and antisepsis of the calvaria. (**B**) U-shaped incision. (**C**) Positioning of the 5 mm diameter cutter to create the defect. (**D**) Exposure of the calvaria defect. (**E**) Insertion of particulate autogenous bone. (**F**) Insertion of Plenum^®^ Osshp. (**G**) Placement of Plenum^®^ Guide over the defect. (**H**) Closure of the tissue with simple sutures and antisepsis.

All animals received 30 mg/kg of ketamine hydrochloride (Syntec-Santana de Parnaíba, SP, Brazil) and 10 mg/kg of xylazine hydrochloride (Syntec-Santana de Parnaíba, SP, Brazil) intramuscularly for anesthesia [18,25]. The comprehensive surgical procedure (Figure 1A–C) and post-operative management have been detailed in previous publications [16,26]. In the AUTOPT+PG group, the parietal bone was removed and ground into smaller pieces to cover the defect created (Figure 1D) [16]. After filling the defect with particulate autogenous bone or Plenum^®^ Osshp (Figure 1E,F), the autogenous bone or biomaterial was covered with the Plenum^®^ Guide membrane (M3 Health Indústria Comércio de Produtos Médicos Odontológicos e Correlatos S.A., Jundiaí, SP, Brazil) exclusively to cover the critical bone defect and keep the biomaterial in place (Figure 1G), followed by primary closure with a 4-0 silk thread (J&J Ethicon^®^, Jardim das Indústrias, São José dos Campos, Brazil) (Figure 1H). Membrane customization is described in detail by Pitol-Palin et al. 2022 [16].

### 2.4. Distribution of Samples and Laboratory Processing

The 48 rats (*n* = 24 per group) were sacrificed with an anesthetic overdose of xylazine hydrochloride and ketamine hydrochloride at 7, 30, and 60 days after calvaria surgery. Of these, *n* = 12 samples were used for immunohistochemistry and histometric analysis at 7 days and 30 days, 6 samples per period; at 30 days, *n* = 6 samples were separated for RT-PCR, and at 60 days after surgery, *n* = 6 samples were used for Micro-CT and confocal analysis.

Every sample used for RT-PCR analysis was collected at the time of euthanasia, stored in 2.0 mL cryogenic tubes (Corning^®^, Corning, NY, USA), and reserved in a freezer at −80 °C (Thermo Scientific, Waltham, MA, USA). The samples for the other analyses were compacted, fixed in 10% formaldehyde (Dinâmica^®^ Química Contemporânea Ltd.a., Indaiatuba, SP, Brazil) for 48 h and, washed in tap water for 24 h.

### 2.5. Microtomographic Analysis (Micro-CT)

**Figure 2:**
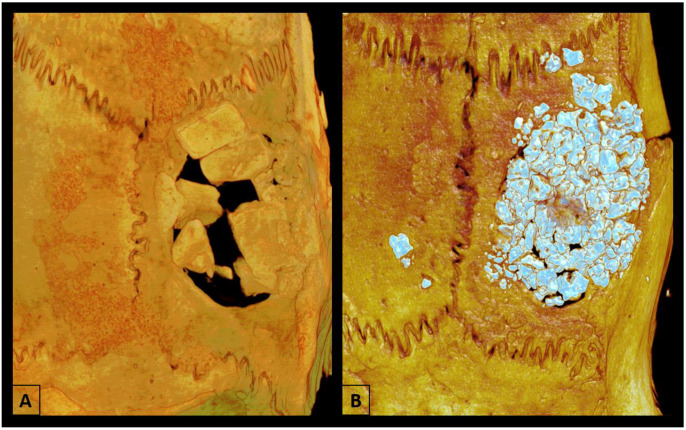
3D images were obtained using CTVox software, showing the bone formation of particulate autogenous bone + Plenum^®^ Guide and Plenum^®^ Osshp + Plenum^®^ Guide. (**A**) AUTOPT+PG group and (**B**) PO+PG group.

The samples were fixed in 70% alcohol and kept for analysis by Micro-CT and confocal laser microscopy after the removal of calvaria at 60 days post-surgery. Micro-CT scanning was performed using a SkyScan 1272 microtomography (SkyScan 1272 BrukerMicroCT, Leuven, Belgium). The images were reconstructed using NRecon software (SkyScan, Leuven, Belgium, 2011; Version 1.6.6.0) and repositioned in three planes (transverse, longitudinal, and sagittal) using Data Viewer software (SkyScan, Leuven, Belgium, Version 1.4.4 64-bit). The defect was evaluated using the CTAnalyser-CTAn software (2003-11SkyScan, 2012 BrukerMicroCT Version 1.12.4.0), and the region of interest (ROI) was determined at a diameter of 5 mm and 30 cuts. The 3D images were obtained using CTVox software (SkyScan, Leuven, Belgium, 2003; Version 3.3.1) (Figure 2) [16]. The percentage of bone volume (BV/TV), bone surface (BV), trabecular thickness (Tb.Th), trabecular number and separation (Tb.N, Tb.Sp), and total porosity (Po.Tot) were determined [27].

### 2.6. Laser Confocal Microscopy Analysis

At 44 and 54 days after calvarial grafting, rats received 20 mg/kg of the fluorochrome calcein and 30 mg/kg of the fluorochrome alizarin. These substances have an affinity for calcium and during the bone repair process, it was possible to measure precipitation in the bone matrix [28]. The samples were collected 60 days after surgery and the entire processing of calvarial pieces for confocal microscopy analysis was described in a previous publication [29]. The final section was 20 μm thick and the samples were then mounted on glass slides with Araldite^®^ Professional epoxy adhesive (Huntsman Advanced Materials, Pirajussara, Taboão da Serra, SP, Brazil). The sections were captured on a Leica Microsystems Stellaris 5 laser confocal microscope (Leica Microsystems Stellaris 5, Heidelberg, Baden-Württemberg, Germany), with a 10× objective (original magnification 100 µm), at the Araçatuba School of Dentistry–UNESP, SP, Brazil).

Thus, fluorochromatic images of calcein (old bone) and alizarin red (new bone) were superimposed to estimate the daily calcium mineral apposition rate on the matrix. The images were evaluated using the ImageJ software (Image Processing and Analysis Software, Bethesda, MD, USA; Java, Version ImageJ 1.53e; the Straight tool was used and the daily mineral apposition (MAR) was measured from five measurements extending from the outer edge of the calcein fluorochrome to the outer edge of alizarin fluorochrome [29]. The total value obtained was divided by the 10-day interval between injections of the two fluorochromes analyzed [30].

### 2.7. Gene Expression (RT-PCR)

Thirty days after the calvaria defect surgery, six animals from each group (AUTOPT+PG and PO+PG) were used to analyze the expression of the following genes: runt-related transcription factor 2 (RUNX2), vascular endothelial factor (VEGF), alkaline phosphatase (ALP), sialoprotein binding to integrin (IBSP), and osteocalcin (OCN). The bone tissue samples were collected using a 7 mm diameter trephine (Harte^®^, Ribeirão Preto, SP, Brazil) under continuous irrigation with phosphate-buffered saline (PBS). Complete RT-PCR processing is detailed in a previous publication [18].

### 2.8. Histometry Analysis (H.E)

After euthanasia, all of the samples were decalcified in 10% EDTA (Exodo^®^ Científica Química Fina Indústria e Comércio Ltd.a., Sumaré, SP, Brazil) for approximately six weeks. The calvarial samples were then fixed in paraffin (Labsynth^®^ Produtos-Laboratório Ltd.a., Diadema, SP, Brazil), sectioned into 6 mm thick coronal sections, mounted on slides, and stained with hematoxylin and eosin for histometric analysis (H.E) at 7 and 30 days and immunolabeling analysis at 30 days.

The slides were scanned (MoticEasyScan One slide scanner, Wetzlar, Hessen, Germany) to scan the entire section (4× magnification) and images were obtained at 10× and 20× magnification. After obtaining the images, a single calibrated analyzer performed the histological description of each of the proposed groups, according to previous studies [16].

### 2.9. Immunolabeling Analysis

For the immunolabeling analysis, runt-related transcription factor 2 (RUNX2), osteopontin (OPN), osteocalcin (OCN), and resistant to tartrate acid phosphatase (TRAP) were used as primary antibodies. These proteins make it possible to evaluate the activity of osteoblastic and osteoclastic cells in different aspects of bone formation, mineralization, and resorption activity. All of the laboratory steps for immunolabeling analysis can be read in a previous publication [18].

Then, after evaluating under a microscope (LeicaR DMLB, Heerbrugg, St. Gallen, Switzerland), scores were determined (ordinal qualitative analysis) by the degree of intensity of labeling with diaminobenzidine (ThermoFisher Scientific, Waltham, MA, USA), defined as discrete (+), moderate (++), and intense (+++) coloration [31,32].

These scores were established according to previous studies and were carried out by a single evaluator (R.O), taking care to keep negative controls to estimate the specificity of the antibodies. When there was no staining, it was considered 0%; light staining represented around 25% of the immunostained area, moderate staining 50%, and intense staining 75% [29,31,33].

### 2.10. Statistical Analysis

Statistical analysis was performed using GraphPad Prism 7.03 software (GraphPad Software, La Jolla, CA, USA). The Shapiro–Wilk test was performed and the normal distribution of the data was confirmed. Thus, the one-way ANOVA test and Tukey’s post-test were applied to determine the differences between the groups, with a significance level of *p* < 0.05.

## 3. Results

### 3.1. Microtomographic Analysis (Micro-CT)

#### 3.1.1. Bone Volume Percentage (BV.TV)

**Figure 3:**
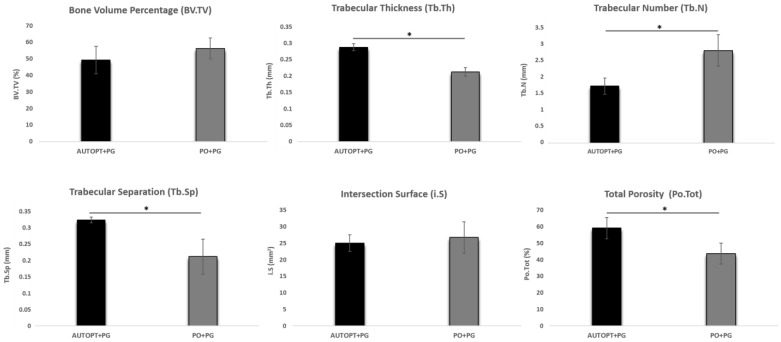
Graphical representation of the results of the Micro-CT analysis: bone volume percentage (BV.TV), trabecular thickness (Tb.Th), trabecular number (Tb.N), trabecular separation (Tb.Sp), intersection surface (i.S), and total porosity (Po.tot). The asterisks in the graphic representation denote statistical differences between groups (*p* < 0.05).

No statistically significant difference was observed between the two groups in terms of bone volume percentage. But, PO+PG (BV.TV = 56.26%) showed a higher percentage of bone volume than the AUTOPT+PG group (BV.TV = 49.29%) (Figure 3 and Table 1).

#### 3.1.2. Trabecular Thickness (Tb.Th)

A statistically significant difference was observed in the comparison between AUTOPT+PG and PO+PG, and the autogenous particulate bone group showed more trabecular thickness (Tb.Th = 0.28 mm) (Figure 3 and Table 1).

#### 3.1.3. Trabecular Number (Tb.N)

The number of trabeculae was higher in the synthetic bone graft group (Tb.N = 2.76 mm), with a statistically significant difference between AUTOPT+PG and PO+PG (Figure 3 and Table 1).

#### 3.1.4. Trabecular Separation (Tb.Sp)

For trabecular separation, there was a statistical difference between AUTOPT+PG and PO+PG, and the AUTOPT+PG group obtained the greatest separation between the bone trabeculae (Tb.Sp = 0.32 mm) (Figure 3 and Table 1).

#### 3.1.5. Intersection Surface (i.S)

The group with the largest intersection surface was the group grafted with biphasic bioceramics of synthetic origin (i.S = 26.76 mm^2^) and there was a statistical difference between AUTOPT+PG and PO+PG (Figure 3 and Table 1).

#### 3.1.6. Total Porosity (Po.Tot)

For total porosity, there was a statistical difference between AUTOPT+PG and PO+PG, and the AUTOPT+PG group showed a higher percentage of total porosity (Po.Tot = 59.15%) (Figure 3 and Table 1).

### 3.2. Laser Confocal Microscopy Analysis

**Figure 4:**
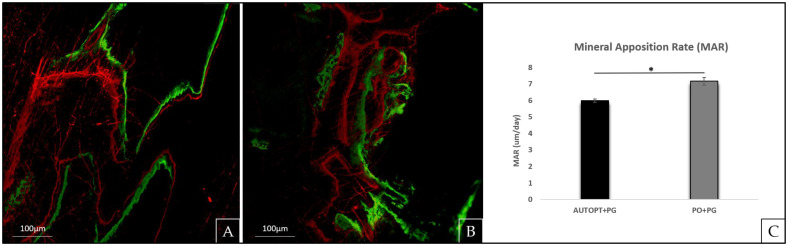
Fluorochrome analysis. Mineral apposition rate (MAR), represent the green (fluorochromes calcein) and red (fluorochromes alizarin). (**A**) AUTOPT+PG group; (**B**) PO+PG group; and (**C**) graph of MAR results. The asterisks in the graphic representation denote statistical differences between groups (*p* < 0.05). Scale bar: 100 µm (10× magnification).

From the laser confocal microscopy analysis, we could evaluate the area of the bone defect created, and it was observed that the PO+PG group obtained a higher daily calcium mineral deposition on the bone matrix 7.16 μm/day (SD: ±0.23) and the AUTOPT+PG group obtained a lower value of 5.99 μm/day (SD: ±0.10), showing a statistical difference between the two groups (*p* = 0.0014, Tukey). (Figure 4C and Table 1).

Figure 4A,B, AUTOPT+PG, and PO+PG groups, respectively, represent the green (fluorochromes calcein) and red (fluorochromes alizarin) injected 10 days apart, marking the calcium matrix, and it is from these images that we can assess daily mineral apposition (MAR) throughout the bone repair period (Figure 4A,B).

### 3.3. Gene Expression Analysis (RT-PCR)

#### 3.3.1. Transcription Factor 2 (RUNX2)

**Figure 5:**
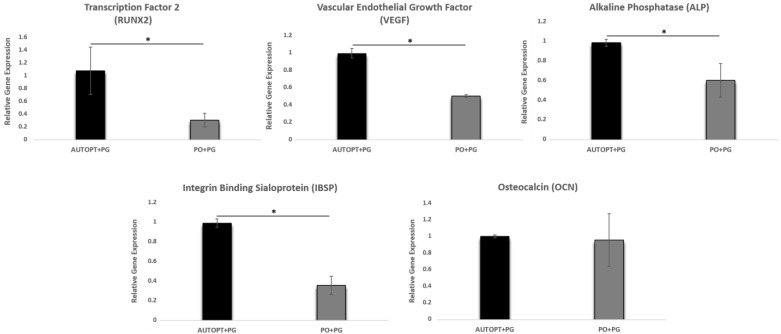
Graphs showing the relative expression of RUNX2, VEGF, ALP, IBSP, and OCN genes for the AUTOPT+PG vs. PO+PG groups. The asterisks in the graphic representation denote statistical differences between groups (*p* < 0.05).

Transcription factor 2 (RUNX2) is related to osteoblast differentiation, and greater expression of this gene was observed in the AUTOPT+PG group (RUNX2 = 1.06), showing a statistically significant difference between the groups (Figure 5 and Table 2).

#### 3.3.2. Vascular Endothelial Growth Factor (VEGF)

There was a statistical difference between the groups (AUTOPT+PG and PO+PG). The highest expression of the VEGF gene was in the group with particulate autogenous bone graft (VEGF = 0.99), which is essential for the growth of vascular endothelial cells (Figure 5 and Table 2).

#### 3.3.3. Alkaline Phosphatase (ALP)

Alkaline phosphatase is a gene responsible for precipitating phosphate in the extracellular matrix, an essential factor in bone mineralization. A statistical difference was observed between the two groups, with the AUTOPT+PG group showing the highest result (ALP = 0.99) (Figure 5 and Table 2).

#### 3.3.4. Osteocalcin (OCN)

There was no statistical difference in the comparison between the groups, and the expressed gene (OCN), responsible for the maturation of osteoblastic cells, had similar results between the autogenous group and the synthetic biomaterial group (Figure 5 and Table 2).

#### 3.3.5. Integrin Binding Sialoprotein (IBSP)

There was a statistical difference between the groups (AUTOPT+PG and PO+PG), with the AUTOPT+PG group showing higher values for the bone sialoprotein gene (IBSP = 0.99), an essential component of bone tissue mineralization (Figure 5 and Table 2).

### 3.4. Histological Analysis

#### 3.4.1. Bone Repair Process at 7 Days

**Figure 6:**
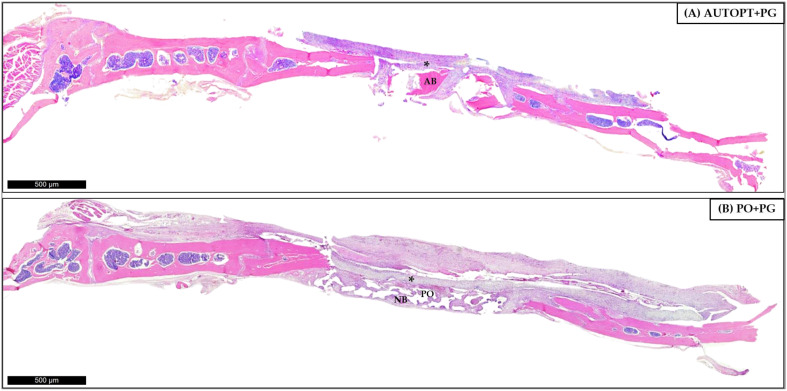
Seven-day histological analysis of groups (**A**) AUTOPT+PG and (**B**) PO+PG. Staining: hematoxylin and eosin. Legend: * Plenum^®^ Guide membrane; AB: autogenous bone; PO: Plenum^®^ Osshp; and NB: neoformed bone. Scale bar: 500 µm (4× magnification).

After euthanasia at 7 days after bone grafting, a panoramic view of the histological slide was taken and it was possible to see particulate autogenous bone (AB) in the center of the defect, arranged in square and pyramidal shapes, surrounded by the right and left bone stumps, covered by the polydioxanone membrane (*), and surrounded by connective tissue with inflammatory cells in greater quantity (Figure 6A).

Figure 6B shows clusters of the biphasic bioceramic graft of synthetic origin (PO) around small islands of neoformed bone (NB) in the center and surrounding connective tissue, with large numbers of inflammatory cells covered by the PDO membrane (*), at its ends we can see the right and left bone stumps.

**Figure 7:**
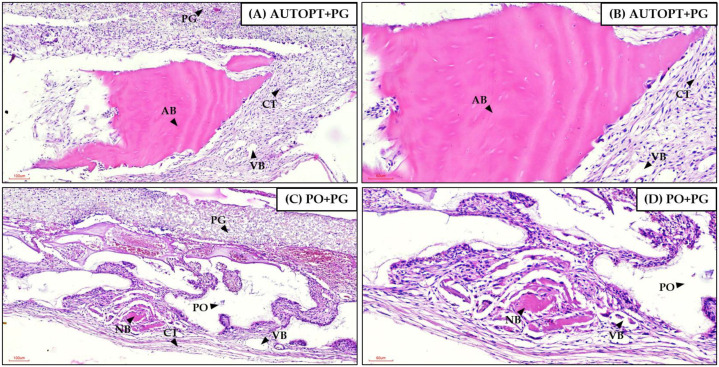
Seven-day histological analysis of groups (**A**,**B**) AUTOPT+PG and (**C**,**D**) PO+PG. Staining: hematoxylin and eosin. Legend: AB: autogenous bone; PO: Plenum^®^ Osshp; NB: neoformed bone; PG: Plenum^®^ Guide; CT: connective tissue; and VB: blood vessel. Scale bar: (**A**,**C**) 100 µm (magnification 10×) and (**B**,**D**) 60 µm (magnification 20×).

Figure 6 was magnified 10× and 20× for better visualization. It was possible to see in the AUTOPT+PG group a pyramidal band of bone tissue with osteoblasts in the matrix and connective tissue organized around it (Figure 7A,B). The PO+PG group also showed recent bone formation, areas of remaining biomaterial replacement, and surrounding connective tissue (Figure 7C,D). The PDO membrane covers the defect in both groups (Figure 7).

#### 3.4.2. Bone Repair Process at 30 Days

**Figure 8:**
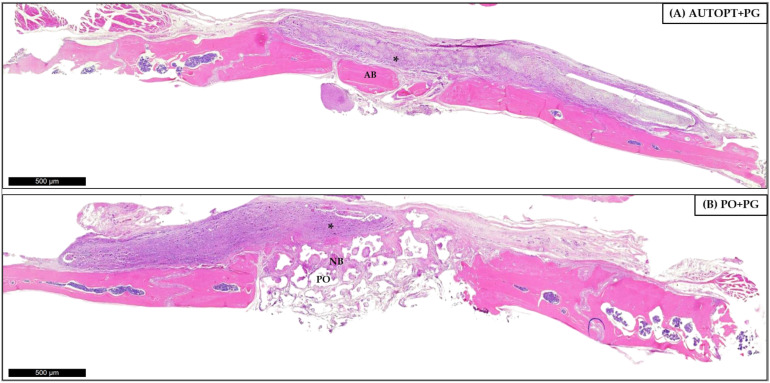
Thirty-day histological analysis of groups (**A**) AUTOPT+PG and (**B**) PO+PG. Staining: hematoxylin and eosin. Legend: * Plenum^®^ Guide membrane; AB: autogenous bone; PO: Plenum^®^ Osshp; and NB: neoformed bone. Scale bar: 500 µm (4× magnification).

For the 30 days, panorama capture was performed on the histological slide and it was possible to observe the progress of the bone repair process when compared to the most recent period, showing bone formation in the areas in the center of the defect where they were filled with autogenous bone or synthetic bone graft; at the ends it is possible to observe the right and left bone stump. Figure 8A shows a cluster of particulate autogenous graft (AB) and surrounding organized connective tissue and the membrane (*) covering the entire defect region (Figure 8A).

The PO+PG group shows small islands of bone tissue (NB) surrounded by organized connective tissue and clusters of synthetic graft (PO) that have not yet been completely resorbed and replaced. The entire defect is covered by the PDO membrane (*) (Figure 8B).

**Figure 9:**
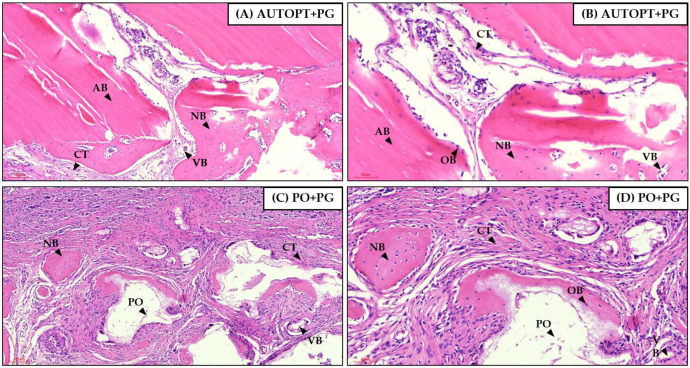
Thirty-day histological analysis of groups (**A**,**B**) AUTOPT+PG and (**C**,**D**) PO+PG. Staining: hematoxylin and eosin. Legend: AB: autogenous bone; PO: Plenum^®^ Osshp; NB: neoformed bone; OB: osteoblast; CT: connective tissue; and VB: blood vessel. Scale bar: (**A**,**C**) 100 µm (magnification 10×) and (**B**,**D**) 60 µm (magnification 20×).

Figure 8 was magnified 10× and 20× for better visualization. In the AUTOPT+PG group, it was possible to see blocks of neoformed bone tissue with osteoblastic cells and organized connective tissue (Figure 9A,B). In the PO+PG group, it was possible to see remnants of the grafted biomaterial surrounded by neoformed bone tissue and well-organized connective tissue (Figure 9C,D).

### 3.5. Immunolabeling Analysis

Immunohistochemical analysis was carried out using the RUNX2, OPN, OCN, and TRAP proteins, and the 30-day period of bone repair was assessed in both groups.

**Figure 10:**
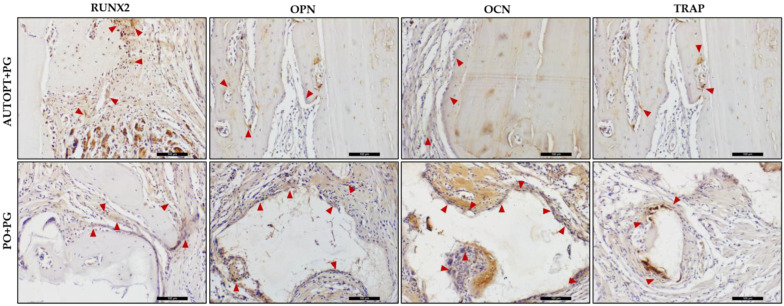
Immunohistochemical analysis of RUNX2, OPN, OCN, and TRAP in the AUTOPT+PG and PO+PG groups 30 days after repair of the critical defect. Contrast stain: Harris hematoxylin; red arrows: immunolabeled cells (represent a positive marker for the observed protein). Scale bar: 100 µm (20× magnification).

The RUNX2 protein is related to the differentiation phase of young osteoblasts and regulates cell proliferation. The AUTOPT+PG group showed moderate labeling (++) for this protein, as did the PO+PG group (Table 3 and Figure 10).

For the OPN protein, the AUTOPT+PG group showed mild labeling (+). For the PO+PG group, expression was moderate (++), so the osteoblasts were arranged in the extracellular matrix, demonstrating the expression of this protein at the start of mineralization in the bone repair area (Table 3 and Figure 10).

The OCN protein is related to the maturation of osteoblasts, an essential phase in the bone mineralization process, with light marking (+) being observed for the AUTOPT+PG group and intense (+++) for the PO+PG group (Table 3 and Figure 10).

In terms of osteoclastic activity, the TRAP protein was lightly labeled (+) for both groups. In the PO+PG group, osteoclastic cell labeling (resorptive activity) was observed near the biomaterial graft area (Table 3 and Figure 10).

## 4. Discussion

Bone tissue grafting is the second most commonly performed transplant procedure worldwide, with more than half a million people in the United States (USA) receiving transplants each year, second only to blood transfusion [34]. Since 1821, when Philip Walter performed the first autogenous bone transplant [35], it is still considered the gold standard for rehabilitating patients with dental problems who need reconstruction in regions of maxillofacial defects [16,36,37]. However, technology has evolved and modernized treatments in the field of oral rehabilitation, and today, the dental industry offers ways of reconstructing areas of extensive bone loss without causing harm to patients, such as bone grafting surgeries with synthetic materials that offer no risk to the patient [11,15,38,39].

Among the options available, the choice for this study was to compare autogenous grafting with a mixture of two compounds widely studied in the literature, hydroxyapatite (HA) and beta-tricalcium phosphate (β-TCP). These two biomaterials manage to play a satisfactory role, as they positively stimulate bone regeneration and form enough bone to cover bone defects [40,41,42]. The concentration of each biomaterial was calculated to favor the best characteristic of both: β-TCP in the proportion of 30% will have slight degradation and will be replaced by neoformed bone quickly, and HA in the concentration of 70%, is not easily resorbed, which keeps the structure integrated for a longer period [7,14,18,43].

Thus, through the results obtained in this study, it was expected that Plenum^®^ Osshp would resemble the gold standard in the area of guided bone regeneration. In the results found from micro-computed tomography, it was possible to observe that there was a statistically significant difference between the AUTOPT+PG vs. PO+PG groups for the parameters of thickness, number of trabeculae, separation of trabeculae, and total porosity, with higher values being noted for the AUTOPT+PG group in all cases. For the percentage of bone volume and intersection surface, there was no statistical difference, with the PO+PG group showing the highest values (BV.TV = 56.26%; i.S = 26.76 mm^2^), respectively, yet the latter group showed lower values for total porosity (Po.Tot = 43.79%) than the particulate autogenous bone group (Po.Tot = 59.15%).

For laser confocal microscopy analysis, the daily mineral apposition rate (MAR), in which the precipitation reaction of calcium on the bone matrix was observed, a statistically significant difference was noted between the two groups, with the group that used the synthetic biomaterial (7.16 µm/day) showing better results than the autogenous group (5.99 µm/day).

These results corroborate findings in the literature [18,44,45], in which it was reported that these synthetic bioceramics in ideal proportions favor osteogenesis, osteoinduction, osteoconduction, and the formation of quality tissue, since β-TCP promotes tissue regeneration by rapid degradation and replacement with new bone and HA maintains the rigid structure for a prolonged time; together, they are ideal for oral rehabilitation when thinking about the installation of osseointegrated implants in areas of critical bone defects [46,47,48,49].

The inflammatory process normally occurs after bone grafting, initially leading to an acute condition in the face of the surgical injury caused. Inflammatory mediators will be released and the bone cells involved will be signaled to the site, where equilibrium will occur after the resorption of the graft and replacement by bone and connective tissue [11].

The expression of RUNX2 is present in pre-osteoblastic cells, which is a characteristic feature of the formation of new bone at the beginning of the inflammatory process since the differentiation and migration of osteoblastic cells to the bone repair region is underway [50,51,52,53,54]. The AUTOPT+PG group showed greater expression of this gene, with a statistical difference when compared to Plenum^®^ Osshp.

The endothelial growth factor, which is derived from osteoblastic cells, regulates the interaction between the production of bone and blood vessels, which is crucial for the development and maintenance of bone health [55,56,57]. A statistical difference was observed between the groups, with greater expression of the VEGF gene in the control group (autogenous bone). The results presented in this study support findings in the literature since biphasic bioceramic agglomerates not yet replaced by bone will have fewer invasions by blood vessels and migration of osteoprogenitor cells to the repair place due to the higher phase of HA (70%) and lower phase of β-TCP (30%) [18,58,59]. Thus, we can assume that the high concentration of hydroxyapatite decreases the rate of the replacement of the biomaterial by bone, which delays the formation of new bone since biological performance is influenced by the particle size of the material and its rate of degradation [18,59,60].

The expression of ALP is related to the beginning of the bone tissue mineralization process, so greater expression was observed in the control group when compared to the bioceramic graft [50]. Bone sialoprotein (IBSP) plays a fundamental role in the formation of bone tissue because, during the repair process, its expression influences the differentiation of stem cells into osteoblastic cells [50,61,62]. The group grafted with autogenous bone showed greater gene expression for new bone formation when likened to the PO+PG group. However, it was detected that once the bone was synthesized, the new bone tissue showed a greater expression of the osteocalcin protein in the group with the synthetic bone graft, and this protein is directly related to the final phase of bone maturation (mineralization) [51].

In the qualitative assessment of bone histometry, it was observed that, for the 7-day repair period after calvaria grafting, there was organized connective tissue and surrounding pyramidal bands of bone tissue with osteoblasts in the matrix for both the AUTOPT+PG group and the PO+PG group in areas of recent bone formation and regions of remaining biomaterial replacement. The polydioxanone membrane covered the entire defect in both groups. At 30 days post-surgery, a much more organized connective tissue could already be seen surrounding the bone tissue, with clusters of blood vessels and islands of newly formed bone with functioning osteoblastic cells in both groups, and remnants of the grafted biomaterial in the PO+PG group.

Immunohistochemical analysis was performed within 30 days. The RUNX2 transcription factor is marked in cells of the osteoblastic lineage, cells that are quite young and in the process of differentiating into active osteoblasts. Therefore, its positive labeling is related to cell renewal at the repair site [52,63]. Moderate labeling for this factor was detected in the AUTOPT+PG group, as well as in the PO+PG group, showing the presence of young cells with potential for osteoblastic differentiation, favoring the repair stages in the region of interest.

Osteopontin, a protein in the extracellular matrix that marks the bone mineralization process [64,65,66], was discreetly marked, especially in the connective tissue present in the region evaluated, and also marked the cementing lines in the mineralized tissue in the AUTOPT+PG group. In the PO+PG group, it was moderately marked in the connective tissue near the biomaterial region. Osteocalcin, a marker of bone mineralization [18,64], was discreet in the connective tissue and present in the mineralized bone tissue in the AUTOPT+PG group. In the PO+PG group, it was intensely marked in regions close to the biomaterial and characterized by bone neoformation.

TRAP-positive osteoclasts mark resorption activity, commanding the actions of the basic multicellular units (BMUs) and the remodeling cycles [16,29,51], and TRAP was discreetly marked in both the AUTOPT+PG and PO+PG groups. It is worth noting that, for the latter, the presence of osteoclasts in resorption activity was observed next to the biomaterial particles present in the defect region.

In this way, we can say that the RT-PCR and histometric analysis were consistent with the results also observed in the immunohistochemical analysis, since from the data collected, we observed that Plenum^®^ Osshp is compatible with the quality of autogenous bone, as neoformed bone tissue was observed around the biomaterial and around organized connective tissue with blood vessels carrying irrigation to the operated area. Positive markings were also observed for the formation, remodeling and mineralization of bone tissue, corroborating statements in the literature in which synthetic bioceramics were used in bone repair [18,58,67].

Consequently, it is expected that the new biphasic synthetic bioceramic (Plenum^®^ Osshp–70/HA:30/β-TCP) could be considered an excellent bone substitute for dentistry since it is believed that this biomaterial is compatible with the gold standard (autogenous bone) because it has properties that promote bone regeneration and characteristics that promote patient safety: it is biocompatible, osteoconductive, osteoinductive, atoxic, and has the ability to induce bone biomineralization.

Therefore, we can conclude that the synthetic bioceramic tested (Plenum^®^ Osshp) provided a satisfactory environment for the stimulation of osteoblastic cells and calcium deposition on the tissue matrix since there was a degradation of the biomaterial remnants and formation of new bone tissue. This leads us to understand that, over time, bone remodeling will replace the implanted bioceramic with vital bone in its entirety, making it a safe and efficient bone substitute for guided bone regeneration in patients with reconstruction needs in bone defects.

## 5. Conclusions

After evaluating all of the results, it can be concluded that the synthetic bone graft (Plenum^®^ Osshp–70:30/HA:β-TCP) covered with polydioxanone membrane (PDO) (Plenum^®^ Guide) proved to be satisfactory in the bone repair process in calvaria defect in rats, being compatible with the gold standard in bone reconstructions (autogenous bone), and an efficient option worldwide as a substitute for bone tissue in bone reconstructions.

## Figures and Tables

**Table 1:** Micro-CT analysis and laser confocal microscopy. Results and standard deviation (SD) for the AUTOPT+PG and PO+PG groups.

	Particulate Autogenous Bone + Plenum^®^ Guide (AUTOPT+PG)	Plenum^®^ Osshp + Plenum^®^ Guide (PO+PG)
**BV.TV (%)**	49.29 ± 8.376 ^a^	56.26 ± 6.245 ^a^
**Tb.Th (mm)**	0.28 ± 0.010 ^a^	0.21 ± 0.013 ^b^
**Tb.N (mm)**	1.69 ± 0.242 ^a^	2.76 ± 0.471 ^b^
**Tb.Sp (mm)**	0.32 ± 0.008 ^a^	0.20 ± 0.052 ^b^
**i.S (mm^2^)**	25.04 ± 2.512 ^a^	26.76 ± 4.736 ^a^
**Po.Tot (%)**	59.15 ± 6.370 ^a^	43.79 ± 6.285 ^b^
**MAR (µm/day)**	5.99 ± 0.105 ^a^	7.16 ± 0.235 ^b^

Statistical differences are denoted by letters (a, b). One-way ANOVA (*p* > 0.05).

**Table 2:** RT-PCR analysis. Representation of the statistical difference and standard deviation (SD) between AUTOPT+PG and PO+PG.

Expression Genes	Particulate Autogenous Bone + Plenum^®^ Guide (AUTOPT+PG)	Plenum^®^ Osshp + Plenum^®^ Guide (PO+PG)
**RUNX2**	1.06 ± 0.365 ^a^	0.30 ± 0.107 ^b^
**VEGF**	0.99 ± 0.056 ^a^	0.50 ± 0.015 ^b^
**ALP**	0.99 ± 0.034 ^a^	0.60 ± 0.172 ^b^
**IBSP**	0.99 ± 0.042 ^a^	0.35 ± 0.094 ^b^
**OCN**	0.99 ± 0.017 ^a^	0.95 ± 0.157 ^a^

Statistical differences are denoted by letters (a, b). One-way ANOVA (*p* > 0.05).

**Table 3:** Immunolabeling scores for the AUTOPT+PG and PO+PG groups at 30 days of bone repair. RUNX2, OPN, OCN, and TRAP antibodies.

	Particulate Autogenous Bone + Plenum^®^ Guide (AUTOPT+PG)	Plenum^®^ Osshp + Plenum^®^ Guide (PO+PG)
**RUNX2**	**++**	**++**
**OPN**	**+**	**++**
**OCN**	**+**	**+++**
**TRAP**	**+**	**+**

The markings are evaluated in the center of the calvaria defect. Mild marking (+), moderate marking (++), and intense marking (+++).

## Data Availability

The data presented in this study are available on request from the corresponding author.
